# Changes in Predominance and Diversity of Genomic Subtypes of *Bordetella pertussis* Isolated in the United States, 1935 to 1999

**DOI:** 10.3201/eid0801.010021

**Published:** 2002-01

**Authors:** Terri Hawes Hardwick, Pamela Cassiday, Robbin S. Weyant, Kristine M. Bisgard, Gary N. Sanden

**Affiliations:** Centers for Disease Control and Prevention, Atlanta, Georgia, USA

**Keywords:** pertussis, pulsed-field gel electrophoresis

## Abstract

Pulsed-field gel electrophoresis (PFGE) of *Bordetella pertussis* chromosomal DNA fragments generated by *Xba*I restriction has been used to subtype isolates for epidemiologic studies. To better understand the natural history of pertussis, we determined the PFGE profiles of 1,333 strains isolated in the United States from 1935 to 1999. Results showed a shift in prevalent profiles from the earliest to the latest study periods. In addition, genetic diversity decreased over time, and prevalent profiles were more highly related to each other than to less common profiles. These results provide the foundation for investigating the impact of prevention strategies, including the use of the acellular vaccines, on the currently circulating *B. pertussis* population.

Pertussis, or whooping cough, is an acute respiratory disease caused by *Bordetella pertussis*. Before pertussis vaccine was introduced in the United States in the mid-1940s, pertussis was a major cause of childhood illness and infant death (*1*). Pertussis incidence decreased after vaccination programs were introduced, and cases were reduced >90% compared with the prevaccination era. Despite vaccine intervention, pertussis remains endemic and epidemic peaks recur every 3 to 5 years (*1*–*8*). Since the early 1980s, the reported incidence of pertussis has increased, especially among adolescents and young adults (*1*,*2*,*4*).

Because vaccination coverage has been continuously high during this period, the observed shift in pertussis epidemiology may be a consequence of pertussis diagnosis and reporting or host factors, such as waning vaccine-induced immunity. A third explanation involves changes in the circulating *B. pertussis* population leading to increased virulence or resistance to vaccine-induced immunity. Differences in *B. pertussis* genetic subtypes circulating in the pre- and post-vaccination eras were demonstrated in the Netherlands, but the role of vaccination in subtype selection remains equivocal (*9*–*11*). Consequently, documentation of the natural history of the U.S. *B. pertussis* population may help explain recent and past changes in pertussis epidemiology and provides the comparative basis for recognizing current and future trends, including those potentially associated with prevention strategies.

Two principal technologies differentiate epidemiologically relevant strains of *B. pertussis*: gene-sequencing analysis and pulsed-field gel electrophoresis of genomic DNA fragments generated by endonuclease restriction (PFGE). In general, sequencing analysis applies to genes with specified relevance, such as virulence determinants, while PFGE subtyping provides a broader perspective of the genome. PFGE subtyping has produced stable and highly reproducible profiles of *B. pertussis* isolates in different laboratories and has sensitively discriminated among epidemiologically distinct isolates (*12*–*15*). Therefore, we used genetic subtyping of recently and previously circulating *B. pertussis* isolates to elucidate the natural history of this pathogen in the United States from 1935 to 1999 and assessed subtypes for temporal trends.

## Material and Methods

### Bacterial Strains

A total of 1,333 *B. pertussis* strains were examined, including a convenience sample of 16 strains from 1935 to 1965 (designated archival); 127 strains from 1966 to 1985 (designated medial); and 1,190 strains from 1986 to 1999 (designated contemporary). Archival and medial isolates were from several different collections. Many of the later contemporary isolates were from enhanced surveillance programs. Overall, 61% of the isolates were from the metropolitan Cincinnati area (n = 480), Massachusetts (n = 205), or Minnesota (n = 125). A second analysis of PFGE profile results excluded the 480 Cincinnati isolates (1989 to 1996) and thus was based on 710 contemporary and 853 total strains. (A table listing all strains and PFGE profiles is available upon request.) Nomenclature of profiles was designated according to Centers for Disease Control and Prevention nomenclature, whereby: CY = *Bordetella*; X = pertussis; XI = *Xba*I; and three number fields identify the PFGE profile. Thus, CYXXI-010 identifies PFGE profile #10 derived from *Xba*I restriction of *B. pertussis* genomic DNA. *B. pertussis* isolates were grown for 3 to 5 days at 37°C on charcoal agar supplemented with 10% defibrinated horse blood under ambient air and high humidity.

### Preparation of Chromosomal DNA

Chromosomal DNA was prepared by following the PFGE protocol published by Gautom (*16*), with modifications. Cells of each strain were harvested after 3 to 5 days' incubation, and strains were suspended in 2 mL of Tris EDTA buffer. The optical density of each cell suspension was adjusted between 0.48 and 0.52 by using a MicroScan turbidity meter (Baxter Diagnostics Inc., Deerfield, IL). Two hundred microliters of each suspension was transferred to 1.5-mL microcentrifuge tubes, and 10 μL of a 20 mg/mL solution of proteinase K (Amresco, Solon, OH) was added. Tubes were gently inverted six times, and 200 μL of the previously described 1.6% InCert agarose/SDS solution was added. The cell and agarose suspensions were mixed without aerosolizing by aspirating and expelling with a 200-μL pipeter and were immediately dispensed into two wells of a 100-μL disposable plug mold (Bio-Rad Laboratories, Hercules, CA) and allowed to solidify at ambient temperature. Plugs were transferred to 2-mL round-bottom microcentrifuge tubes containing 1.5 mL of the previously described EDTA/Sarcosine buffer and 40 μL of a 20-mg/mL solution of proteinase K. Tubes were immersed horizontally in a reciprocal shaking water bath (130 strokes per minute) at 50°C and incubated for 1.5 hours. The plugs were then transferred to 50-mL conical centrifuge tubes and washed with 10 mL of sterile distilled water for 15 minutes, then five times in 10 mL of the previously described Tris EDTA buffer for 30 minutes each. For each wash, tubes were placed in a reciprocal shaking water bath at 50°C and 150 strokes per minute. Plugs were stored and maintained in fresh plug wash buffer at 4°C for up to 4 months before restriction endonuclease digestion.

### Restriction Endonuclease Digestion and PFGE

Two 1-mm slices of the prepared plugs were cut with a sterile razor blade and transferred to 1.5-mL microcentrifuge tubes containing 100 μL of *Xba*I digestion solution (30 U of *Xba*I in the manufacturer’s supplied buffer; Roche Diagnostics, Indianapolis, IN). Plug slices were incubated at 37°C for 1.5 hours. One plug slice of each strain and three slices of a phage lambda molecular weight standard (PFGE Marker I; Roche Diagnostics) were placed on the teeth of a 15-well comb and allowed to adhere for 15 minute at ambient temperature. Molten 1% agarose in 0.5X Tris-Borate-EDTA buffer (TBE; Gibco BRL, Rockville, MD), cooled to 50°C, was poured and allowed to solidify around the comb. The comb was removed and the resulting wells were filled with agarose. PFGE was conducted in 0.5X TBE buffer in a contour-clamped homogenous electric field apparatus (CHEF-Mapper or CHEF-DR III; Bio-Rad). Electrophoresis was performed at 6V/cm for 18 hours at 14°C with a ramped switch time of 2.16 seconds to 35.07 seconds. Gels were stained for 25 minutes with gentle rotation in 250 mL of deionized water containing 25 μL of a 10 mg/mL solution of ethidium bromide. Unbound ethidium bromide was removed by washing gels in deionized water 3 times for 30 minutes each. DNA fragments were visualized on a UV transilluminator, and gels were photographed. The photographic negatives were scanned, and the DNA fragments were analyzed with Diversity Database software (Bio-Rad) with at least one band discrepancy as the basis for discriminating among profiles. *B. pertussis* DNA fragment sizes were determined from their motilities relative to the phage lambda molecular weight standard.

### Statistical Analysis

We calculated genotypic diversity (GD) among profiles as follows: GD=[n/(n-1)](1-3x_i_^2^ ), where x_i_ is the frequency of the i^th^ PFGE profile and n is the total number of *B. pertussis* strains. Values of GD range from 0 to 1, with “0" defined as a population of isolates demonstrating identical profiles and “1” representing a unique profile for each isolate. The statistical significance of differences in GD values was evaluated by computer simulation as described previously (*11*). A PFGE profile relatedness diagram was constructed based on the unweighted pair-group method with arithmetic mean (UPGMA).

## Results

**Figure 1:**
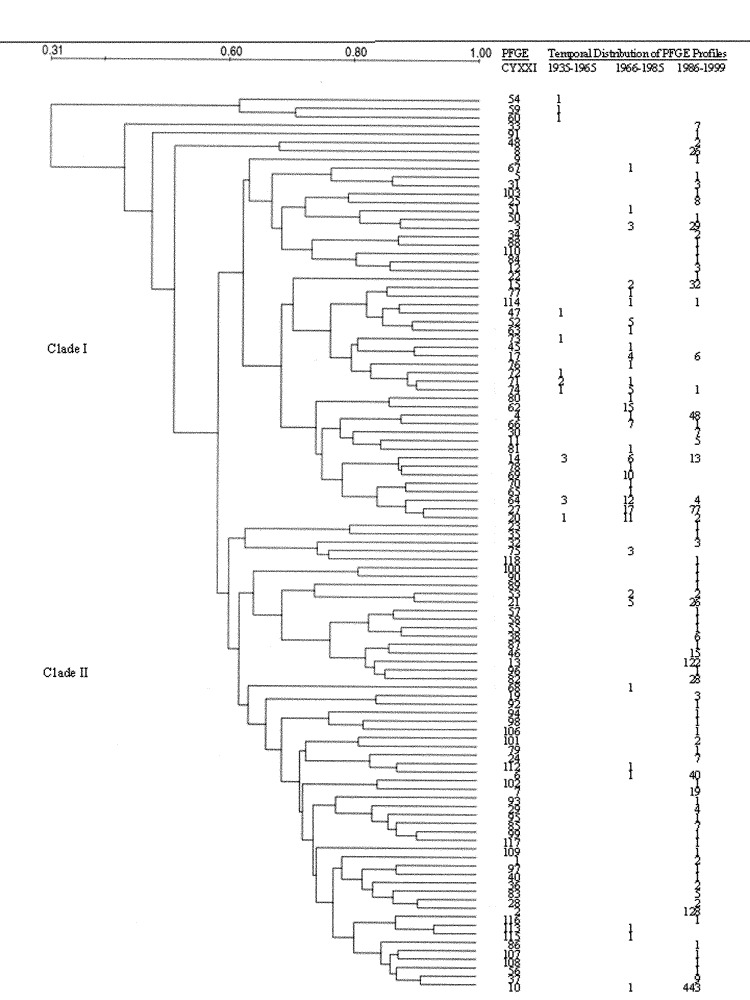
Relatedness of pulsed-field gel electrophoresis profiles of *Bordetella pertussis* strains isolated in the United States,1935 to 1999.

We identified 105 PFGE profiles among the 1,333 *B. pertussis* isolates collected in the United States from 1935 to 1999 (Figure 1). We attempted to assess the geographic distributions of identified profiles in the United States. However, the geographic distribution of archival and medial isolates was limited by isolate collections available, which underrepresented certain regions. Thus, geographic trends in PFGE profile prevalences were not discernible.

**Figure 2:**
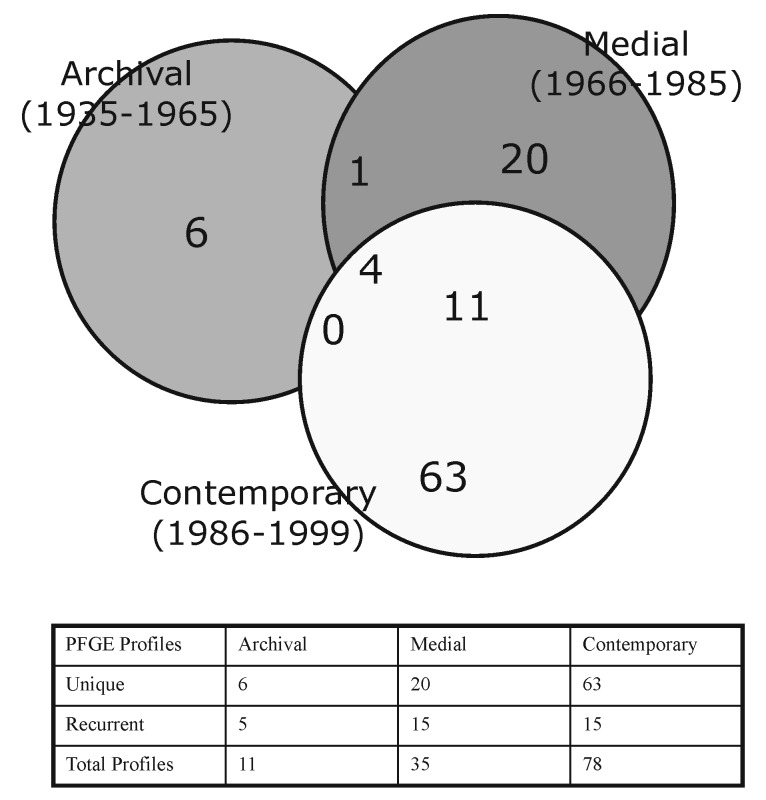
Temporal distribution of 105 pulsed-field gel electrophoresis profiles of *Bordetella pertussis* strains isolated in the United States, 1935 to 1999.

We also evaluated the temporal distribution of isolate profiles (Figures 1 & 2). Eleven, 35, and 17 profiles were observed among 16 archival, 127 medial, and 1,190 contemporary isolates, respectively. Specific profiles tended to be restricted to isolates circulating during a given time period: only 5 (CYXXI-014, -020,-064, -071, and -074) of the 11 archival isolate profiles were also detected in strains circulating in the medial period; and 15 (CYXXI-003, -004, -006, -010, -014, -015, -017, -020, -021, -027, -053, -064, -066, -074, and -114) of the 35 medial profiles were shared by contemporary isolates. Moreover, only four PFGE profiles (CYXXI-014, -020, -064, and -074) were identified in 62 isolates cultured in all three time intervals (Figure 2). Profiles recovered from more than one time period tended to occur in low frequency in later periods. We observed only 4 of 16 frequencies >5% in a subsequent period: CYXXI-020 and CYXXI-064 were seen in 8% and 9% of medial isolates, respectively, and CYXXI-010 and CYXXI-027 were found in 37% and 6% of contemporary isolates, respectively.

The most frequent profiles overall (CYXXI-010, -002, -013, and -027) represented 33%, 10%, 9%, and 7%, of study isolates, respectively (Table). However, the most frequent profiles in any given time period varied. The most frequent archival profiles, CYXXI-014 (19%) and CYXXI-064 (19%), collectively represented more than one third of the strains isolated before 1966. As noted previously, the CYXXI-064 profile continued into the medial period and was the third most prevalent profile after CYXXI-027 (13%) and CYXXI-062 (12%). The three most prevalent contemporary PFGE profiles (CYXXI-010, -002, and -013) represented approximately 58% of the strains isolated since 1986. Profile CYXXI-082 emerged in 1997 and has rapidly increased in frequency to account for 9% of the *B*. *pertussis* isolates from 1997 to 1999.

**Table:** Frequencies of the most prevalent pulsed-field gel electrophoresis (PFGE) profiles of *Bordetella pertussis* strains isolated in the United States from 1935 to 1999

PFGE	Total US n=1,333	Total US w/o Cinn n=853	1935-1965 n=16	1966-1985 n=127	1986-1999 n=1,190	1986-1999 w/o Cinn n=710
2	9.6	4.3	-	-	10.8	5.4
3^a^	2.4	1.3	-	2.4	2.4	1.1
4^a^	3.7	0.4	-	0.8	4	0.3
6^a^	3.1	0.1	-	0.8	3.4	0.1
7	1.4	1.8	-	-	1.6	2.1
8	2	-	-	-	2.2	-
10^a^	33.3	33.9	-	0.8	37.3	40.8
13	9.2	14.1	-	-	10.3	17
14^b^	1.6	2.6	18.8	4.7	1.1	1.8
15^a^	2.6	0.8	-	1.6	2.7	0.8
17^a^	0.8	0.8	-	3.1	0.5	0.4
20^b^	1	1.5	6.3	8.7	0.2	0.1
21^a^	2.3	2.2	-	3.9	2.2	2
27^a^	7.1	9.3	-	13.4	6.5	8.8
37	0.7	1	-	-	0.8	1.3
46	1.1	1.8	-	-	1.3	2.2
52	0.4	0.6	-	3.9	-	-
53^a^	0.3	0.5	-	1.6	0.2	-
62	1.1	1.8	-	11.8	-	-
64^b^	1.4	2.2	18.8	9.4	0.3	0.6
66^a^	0.6	0.9	-	5.5	0.1	0.1
69	0.8	1.2	-	7.9	-	-
71^c^	0.2	0.4	12.5	0.8	-	-
74^b^	0.5	0.8	6.3	3.9	0.1	0.1
75	0.2	0.4	-	2.4	-	-
82	2.1	3.3	-	-	2.4	4
Total	89.5	88	62.7	87.4	90.4	89

^a^PFGE profile spans second and third time periods.

^b^PFGE profile spans all three time periods.

^c^PFGE profile spans first and second time periods.

CINN = Cinncinnati, Ohio, data.

We evaluated the similarities between PFGE profiles of *B. pertussis* isolates and recognized two major relatedness clusters (clades) that were 58% similar (Figure 2). Seven additional PFGE profiles were notable for their divergence (<50% similar). The three profiles (CYXXI-054, CYXXI-059, CYXXI-060) representing the three earliest isolates from 1935 and 1939 were only 31% similar to Clades I and II. Interestingly, contemporary isolates yielded the remaining four divergent profiles (CYXXI-008, -033, -048, and -091). The eight nondivergent profiles from the archival isolates formed a Clade I subgroup with 68% similarity. PFGE profiles from the medial isolates were unevenly distributed in both clades: 60% of Clade I profiles and 16% of Clade II profiles represented 87% and 13% of the medial isolates, respectively. The medial profiles in Clade I were more similar to each other (89% were 68% similar) than those in Clade II. Although contemporary profiles were highly divergent, most (68%) resided in Clade II and represented 78% of these isolates.

A 62% similarity subgroup of Clade II profiles included the three most frequent PFGE profiles during the contemporary period and overall (CYXXI-002, -010, and -013). The most frequent archival (CYXXI-014 and -064) and the most frequent medial (CYXXI-027 and -062) profiles shared a 74% similarity subgroup within Clade I. Two of these predominant profiles (CYXXI-014 and -064) represented isolates from all three time periods, as did profile CYXXI-020, which was also in this relatedness subgroup. The fourth persistent profile, CYXXI-074, was also in Clade I, but not within this 74% similarity subgroup.

The overall GD among the study isolates was 0.86. The GD was 0.94 for the archival strains and 0.94 for the medial strains. The GD decreased to 0.82 for contemporary strains. GD among isolates from the period before vaccine intervention, 1935 to 1946, was 1.00 and decreased to 0.91 for the remainder of the archival strains isolated from 1955 to 1965. Only the decrease in GD between the medial (GD = 0.94) and contemporary (GD = 0.82) periods was significant (p<0.001).

We analyzed PFGE profile data, excluding the 480 contemporary isolates from Cincinnati (*6*), to evaluate how a large source of isolates might affect our results (Table). With this exclusion, 710 contemporary and 853 total isolates were tested. Because 9 of 79 profiles representing contemporary isolates were unique to Cincinnati (CYXXI-005, -008, -009, -022, -023, -025, -030, -032, -035), a total of 96 PFGE profiles, including 70 from contemporary isolates, were included in the analysis. The prevalence of the predominant contemporary profile CYXXI-010 did not change from approximately 33%. Conversely, the overall prevalencies of two other predominant profiles were affected, as CYXXI-002 prevalence decreased from 10% to 4% and CYXXI-013 increased from 9% to 14%. Excluding Cincinnati isolates profiles, the total GD decreased slightly from 0.86 to 0.84, as did the GD for the contemporary isolates (1986 to 1999) from 0.82 to 0.79.

## Discussion

There has been a resurgence of pertussis since the early 1980s in countries with high vaccination rates, including Australia (*17*), Canada (14), Denmark (*18*), the Netherlands (*9*–*11*,*19*), and the United States. Because this resurgence may represent changes in the etiologic agent, we determined the PFGE profiles of *B. pertussis* isolates circulating in the United States between 1939 and 1999 and evaluated these results for any trends with potential epidemiologic significance. We recognized several major trends: different profiles circulated and predominated at different times, the relatedness among the PFGE profiles was consistent with a relatively homogeneous population, the more frequent profiles were more highly related to each other than to less common profiles, and GD decreased over the study period.

The observed tendency of different profiles to circulate in different periods was also reported from the Netherlands, where 83% of DNA types were limited to single periods and only one type was cultured in all successive periods *(11)*. Because PFGE profiles tended to be confined to a single time period, different profiles predominated in different periods. The two most predominant profiles (CYXXI-010 and -002) in the contemporary period emerged in the mid-1980s, temporally dissociated with changes in intervention programs such as vaccination, but coincident with an increasing incidence of pertussis (*4*). A shift in the frequency of allelic types of two immunogenic proteins, pertactin and pertussis toxin subunit A, was also noted during this period, and the new allelic types were strongly associated with isolates having the CYXXI-010 and -002 profiles (*20*). Additional study is needed to determine the role of the newly predominant allelic types in conferring predominance to these PFGE profiles, especially their distribution among less frequent profiles.

Our most dominant (33%) profile (CYXXI-010) is analogous to Canadian profile (a), which represented 34% of all Canadian isolates, suggesting that this profile is widely circulating in North America. We also noted that PFGE profile CYXXI-082 emerged in 1997 but still accounted for >2% of the contemporary isolates, confirming the dynamic nature of the population and suggesting that alternative profiles may predominant in the near future.

We determined PFGE profile similarities and evaluated them for potential associations, especially with predominance or persistence. No two of the 105 profiles were >93% similar, and two major similarity groups, or clades, were identified. The *B. pertussis* population studied in the Netherlands showed similar organization (*11*). This relatedness structure most likely represents the relatively homogeneous or clonal nature of most pathogenic bordetellae, as previously suggested by multilocus enzyme electrophoresis (*21*), DNA polymorphism studies (*22*), and earlier characterizations of strains by PFGE profiling (*15*,*23*). The distribution of several PFGE patterns over all study periods and multiple locations is also consistent with a homogeneous population. In general then, the observed *B. pertussis* population structure likely represents adaptation to a single host species and limited opportunity for horizontal genetic exchange, although such exchange between *B. pertussis* and *B. parapertussis* is evident (*22*,*24*).

The most prevalent profiles overall and in the median period tended to be relatively highly related to each other, suggesting that they may share common, but unknown, genetic characteristics that confer dominance. The four persistent profiles that were observed in isolates from all time periods clustered within Clade I with 68% similarity, suggesting that they may share common properties for longitudinal transmission and that these properties are different and unlinked from elements conferring highest prevalence.

The similarity decreased between profiles from archival and successive periods, and the most predominant profile in the contemporary period showed the greatest divergence. In particular, the PFGE profiles from the prevaccine era (before 1946) isolates were highly divergent from the postvaccination isolate profiles. Others have proposed that the pre- versus postvaccination era population changes may have been driven by selective pressure of whole-cell vaccines; the divergence in our study is consistent with this proposal (*9*–*11*). However, the role of vaccine selection in the divergence observed in our study remains speculative because a relatively low number of isolates was available from the archival period, GD did not decrease significantly until the contemporary period, and similarly divergent PFGE profiles were circulating in the contemporary period.

The divergence among profiles from contemporary isolates was comparatively high, although the GD was lowest for this period. A previous study indicated that increased recovery of isolates led to detection of PFGE subtypes circulating at low frequency (*25*). This suggests that the relatively large number of contemporary isolates permitted identification of infrequent, but divergent profiles not discernible in isolates from the other two periods. Despite this observed divergence and the diversity it implies, the frequency of predominant contemporary PFGE subtypes was sufficiently high to depress the GD relative to the previous periods.

The GD (0.86) of the *B. pertussis* population studied resulted from the prevalence of only a few PFGE profiles, combined with the low numbers of isolates represented by most of the profiles. Investigations in Canada (*14*), the Netherlands (*11*), Italy (*26*), and Mexico (*27*) showed a similar tendency: a few genomic profiles dominated and all others were observed infrequently. Published GD values were also similar in Dutch (*11*) and Mexican (*27*) studies, so our observations may be characteristic for *B. pertussis* populations.

GD was consistent between the archival and the medial isolates, but decreased significantly for the contemporary strains. This reflects relatively fewer profiles representing a greater proportion of contemporary isolates. In contrast, other investigators found a significant decrease in GD in the period after introduction of the whole-cell vaccine. Because the size of our archival time period may have precluded our ability to distinguish a temporally relevant shift in GD, we also calculated GD among isolates from early (1935 to 1955) and late (1956 to 1965) in this period. However, the change in GD was relatively modest, from 1.00 to 0.93.

Evaluating the population structure of *B. pertussis* requires sampling isolates representative of the natural distribution of circulating strains. Three sampling artifacts were inherent in our study. First, the numbers and geographic distribution of archival and medial isolates were limited by the isolate collections available so that the number of contemporary isolates was comparatively much greater. Second, most of the contemporary isolates were from an enhanced surveillance project comprising only six sites (in Arizona, Georgia, Illinois, Massachusetts, Minnesota, and New York). Consequently, the restricted geographic distribution of available isolates precluded evaluating our results for relevant geographic trends. However, the isolation of several prevalent profiles, such as CYXXI-010, from multiple sites in the United States and Canada was consistent with an essentially clonal population (*21*).

Third, an outbreak investigation such as occurred in Cincinnati from 1991 to 1996 and Delaware in 1986 can contribute a disproportionate number of isolates from a local area over a short time because they are more likely to be recovered and retained. To investigate the impact of this bias, we calculated results with and without the Cincinnati PFGE profile data and found that these results did change the prevalences of two of the three predominant profiles, but not GD. Thus, regional differences in profile frequencies apparently exist; future work will attempt to confirm this. However, the impact of epidemic strains did not seem to extend to the general population structure, as defined by GD. Epidemic isolates may limit PFGE profile diversity, especially if clonal expansion had a major role in causing the epidemic. However, we and others have shown that this is not the usual case with pertussis epidemics in general and in the Cincinnati epidemic in particular (*11*,*14*,*24*).

This study is the first to provide data about the distribution of PFGE profiles among the U.S. *B. pertussis* population over an extended period of time. Our results, together with pertussis surveillance data, can serve as the comparative basis for evaluating the potential impact of current and future prevention strategies, including the use of acellular vaccines, on the circulating *B. pertussis* population.

## References

[1] Centers for Disease Control and Prevention. Pertussis-- United States, January 1992-June 1995. MMWR Morb Mortal Wkly Rep. 1995;21:40–4.

[2] Centers for Disease Control and Prevention. Resurgence of pertussis--United States, 1993. MMWR Morb Mortal Wkly Rep 1993;42:952-3,959-60. 8246860

[3] Bass JW, Wittler RR. Return of epidemic pertussis in the United States. Pediatr Infect Dis J. 1994;13:343–5. 10.1097/00006454-199405000-00002 8072813

[4] Guris D, Strebel PM, Bardenheier B, Brennan M, Tachdjian R, Finch E, Changing epidemiology of pertussis in the United States: increasing reported incidence among adolescence and adults, 1990-1996. Clin Infect Dis. 1999;28:1230–7. 10.1086/514776 10451158

[5] Fine PE, Clarkson JA. Reflections on the efficacy of pertussis vaccines. Rev Infect Dis. 1987;9:866–83. 3317732 10.1093/clinids/9.5.866

[6] Christie CDC, Marx ML, Marchant CD, Reising SF. The 1993 epidemic of pertussis in Cincinnati--resurgence of disease in a highly immunized population of children. N Engl J Med. 1994;331:16–21. 10.1056/NEJM199407073310104 8202096

[7] Kenyon TA, Izurieta H, Shulman ST, Rosenfield E, Miller M, Daum R, Large outbreak of pertussis among young children in Chicago, 1993: investigation of potential contributing factors and estimation of vaccine effectiveness. Pediatr Infect Dis J. 1996;15:655–61. 10.1097/00006454-199608000-00004 8858667

[8] Centers for Disease Control and Prevention. Pertussis outbreak--Vermont, 1996. MMWR Morb Mortal Wkly Rep. 1997;46:822–6. 9310216

[9] Mooi FR, van Oirschot H, Heulvelman K, van der Heide HJG, Gaastra W, Willems RJL. Polymorphism in the *Bordetella pertussis* virulence factors P.69/pertactin and pertussis toxin in The Netherlands: temporal trends and evidence for vaccine-driven evolution. Infect Immun. 1998;66:670–5. 9453625 10.1128/iai.66.2.670-675.1998 PMC107955

[10] van Loo IHM, van der Heide HGJ, Nagelkerke NJD, Verhoef J, Mooi FR. Temporal trends in the population structure of *Bordetella pertussis* during 1949-1996 in a highly vaccinated population. J Infect Dis. 1999;179:915–23. 10.1086/314690 10068587

[11] van der Zee A, Vernooij S, Peeters M, van Embden J, Mooi FR. Dynamics of the population structure of *Bordetella pertussis* as measured by IS 1002-associated RFLP: comparison of pre- and post-vaccination strains and global distribution. Microbiology. 1996;142:3479–85. 9004510 10.1099/13500872-142-12-3479

[12] Arbeit RD. Laboratory procedures for the epidemiologic analysis of microorganisms. In: Murray PR, Baron EJ, Pfaller MA, Tenover FC, Yolken RH, editors. Manual of clinical microbiology. 6th ed. Washington: American Society of Microbiology Press; 1995. p.190-208.

[13] Beall B, Cassiday PK, Sanden GN. Analysis of *Bordetella pertussis* isolates from an epidemic by pulsed-field gel electrophoresis. J Clin Microbiol. 1995;33:3083–6. 8586677 10.1128/jcm.33.12.3083-3086.1995 PMC228648

[14] DeMoissac YR, Ronald SL, Peppler MS. Use of pulsed-field gel electrophoresis for epidemiological study of *Bordetella pertussis* in a whooping cough outbreak. J Clin Microbiol. 1994;32:398–402. 8150949 10.1128/jcm.32.2.398-402.1994 PMC263043

[15] Khattak MN, Matthews RC. Genetic relatedness of *Bordetella* species as determined by pulsed-field gel electrophoresis. Int J Syst Bacteriol. 1993;43:659–64. 8240949 10.1099/00207713-43-4-659

[16] Gautom RK. Rapid pulsed-field gel electrophoresis protocol for typing of *Escherichia coli* O157:H7 and other gram-negative organisms in one day. J Clin Microbiol. 1997;35:2977–80. 9350772 10.1128/jcm.35.11.2977-2980.1997 PMC230100

[17] Cordova SP, Gilles MT, Beers MY. The outbreak that had to happen: *Bordetella pertussis* in north-west western Australia in 1999. Commun Dis Intell. 2000;24:375–9. 11225380 10.33321/cdi.2000.24.67

[18] Nielsen A, Larsen SO. Epidemiology of pertussis in Denmark: The impact of herd immunity. Int J Epidemiol. 1994;23:1300–8. 10.1093/ije/23.6.1300 7721534

[19] de Melker HE, Conyn-van Spaendonck MAE, Rumke HC, van Wijngaarden JK, Mooi FR, Schellekens JFP. Pertussis in the Netherlands: an outbreak despite high levels of immunization with whole-cell vaccine. Emerg Infect Dis. 1997;3:175–8. 9204299 10.3201/eid0302.970211 PMC2627603

[20] Cassiday PK, Sanden GN, Heuvelman K, Mooi FR, Bisgard KM, Popovic T. Polymorphism in *Bordetella pertussis* pertactin and pertussis toxin virulence factors in the United States, 1935-1999. J Infect Dis. 2000;182:1402–8. 10.1086/315881 11023463

[21] Musser JM, Bemis BA, Ishikawa H, Selander RK. Clonal diversity and host distribution in *Bordetella bronchiseptica.* J Bacteriol. 1987;169:2793–803. 3584070 10.1128/jb.169.6.2793-2803.1987 PMC212186

[22] van der Zee A, Groenendijk H, Peeters M, Mooi FR. The differentiation of *Bordetella parapertussis* and *Bordetella pertussis* from humans and animals as determined by DNA polymorphism mediated by two different insertion sequence elements suggests their phylogenetic relationship. Int J Syst Bacteriol. 1996;46:640–7. 8782670 10.1099/00207713-46-3-640

[23] Khattak M, Matthews RC, Burnie JP. Is *Bordetella pertussis* clonal? BMJ. 1992;304:813–5. 10.1136/bmj.304.6830.813 1392709 PMC1881643

[24] Smith JM, Smith NH, O’Rourke M, Spratt BG. How clonal are bacteria? Population Biology. 1993;90:4384–8. 10.1073/pnas.90.10.4384 PMC46515 8506277

[25] Bisgard KM, Christie CD, Reising SF, Sanden GN, Cassiday PK, Gomeresall C, Molecular epidemiology of *Bordetella pertussis* by pulsed-field gel electrophoresis profile: Cincinnati, 1989-1996. J Infect Dis. 2001;183:1360–7. 10.1086/319858 11294667

[26] Mastrantonio P, Spigaglia P, van Oirschot H, van der Heide HJG, Heuvelman K, Stefanelli P, Antigenic variants in *Bordetella pertussis* strains isolated from vaccinated and unvaccinated children. Microbiology. 1999;145:2069–75. 10.1099/13500872-145-8-2069 10463173

[27] Cassiday PK, Sapian LA, Guris D, Sanden GN. Genotypic analysis of *Bordetella pertussis* isolates from Mexico. [Abstract P-4.13.] International Conference on Emerging Infectious Diseases, March 1998. Atlanta: Centers for Disease Control and Prevention; 1998.

